# Serum lipidomics profiles reveal potential lipid markers for prediabetes and type 2 diabetes in patients from multiple communities

**DOI:** 10.3389/fendo.2022.966823

**Published:** 2022-08-15

**Authors:** Qiuhui Xuan, Chunxiu Hu, Yinan Zhang, Qingqing Wang, Xinjie Zhao, Xinyu Liu, Congrong Wang, Weiping Jia, Guowang Xu

**Affiliations:** ^1^ Chinese Academy of Sciences (CAS) Key Laboratory of Separation Sciences for Analytical Chemistry, Dalian Institute of Chemical Physics, Chinese Academy of Sciences, Dalian, China; ^2^ Department of Endocrinology, Shandong Provincial Hospital, Shandong University, Jinan, China; ^3^ University of Chinese Academy of Sciences, Beijing, China; ^4^ Shanghai Diabetes Institute, Shanghai Key Laboratory of Diabetes Mellitus, Shanghai Clinical Center for Endocrine and Metabolic Diseases, Metabolic Diseases Biobank, Shanghai Jiao Tong University Affiliated Sixth People’s Hospital, Shanghai, China; ^5^ Department of Endocrinology and Metabolism, Shanghai Fourth People’s Hospital, Tongji University School of Medicine, Shanghai, China

**Keywords:** diabetes, subtypes of prediabetes, impaired glucose tolerance, impaired fasting glucose, lipidomics, dyslipidemia, biomarkers

## Abstract

**Objective:**

Dyslipidemia is a hallmark of diabetes mellitus (DM). However, specific lipid molecules closely associated with the initiation and progression of diabetes remain unclear. We used a pseudotargeted lipidomics approach to evaluate the complex lipid changes that occurred long before the diagnosis of type 2 diabetes mellitus (T2DM) and to identify novel lipid markers for screening prediabetes mellitus (PreDM) and T2DM in patients from multiple communities.

**Methods:**

Four hundred and eighty-one subjects consisting of T2DM, three subtypes of PreDM, and normal controls (NC) were enrolled as discovery cohort. Serum lipidomic profiles of 481 subjects were analyzed using an ultrahigh performance liquid chromatography-triple quadrupole mass spectrometry (UHPLC-QqQ-MS)-based pseudotargeted lipidomics method. The differential lipid molecules were further validated in an independent case-control study consisting of 150 PreDM, 234 T2DM and 94 NC.

**Results:**

Multivariate discriminative analyses show that lipidomics data have considerable potential for identifying lipidome differences among T2DM, subtypes of PreDM and NC. Statistical associations of lipid (sub)species display significant variations in 11 lipid (sub)species levels for T2DM and distinctive differences in 8 lipid (sub)species levels between prediabetic and normoglycemic individuals, with further differences in 8 lipid (sub)species levels among subtypes of PreDM. Adjusted for sex, age and BMI, only two lipid (sub)species of fatty acid (FA) and phosphatidylcholine (PC) were associated at *p*< 0.05 for PreDM (all) and subtypes of PreDM. The defined lipid markers not only significantly improve the diagnostic accuracy of PreDM and T2DM but also effectively evaluating the risk of developing into each subtype of PreDM and T2DM when addition of age, sex, BMI, and FPG, respectively.

**Conclusions:**

Our findings improve insights into the lipid metabolic complexity and interindividual variations among subtypes of PreDM and T2DM, beyond the well-known differences in dyslipidemia in clinic.

## Introduction

Type 2 Diabetes mellitus (T2DM) is one of the most prevalent endocrine diseases worldwide characterized by dyslipidemia and dysglycemia. Prediabetes mellitus (PreDM) is a state of dysglycemia that precedes the onset and development of T2DM (1). The International Diabetes Federation (IDF, 2019) estimates that 700.2 million adults aged 20-79 years will suffer from T2DM by 2045. Currently, the prevalence of PreDM in adults is reported to be 38% in the USA and 35.7% % in China (2, 3). T2DM and PreDM have become one of the leading challenges of public health in the world.

Unfortunately, most diabetic patients have no obvious disease symptoms in the early stage, leading to miss the opportunities for timely diagnosis of T2DM. Individuals with PreDM experience a certain degree of lipid metabolic disorder and are likely to develop overt T2DM (4). PreDM can be divided into three different subcategories such as impaired glucose tolerance (IGT), impaired fasting glucose (IFG), and IGT plus IFG from an oral glucose tolerance test (OGTT) data according to World Health Organization (WHO 1999) diagnostic criteria. Although all three belong to PreDM, they differ somewhat in their biological underpinnings. For example, fasting and 2-h glucose differ in hepatic insulin sensitivity, muscle insulin resistance and first- and second-phase insulin responses (5). Furthermore, a few of those with PreDM are so identified on all three subcategories (6).

Conventional diabetic risk factors such as plasma triacylglycerols (TG), total cholesterol (TC), small dense low density lipoprotein cholesterol (LDL-C), and high-density lipoprotein cholesterol (HDL-C), are well-established (7–9). However, it is not well understood whether independent pathways exist that bypass these conventional factors altogether due to large individual differences and the complexity of the pathophysiological mechanisms of diabetes. To this end, further understanding the lipid molecular pathways underlying prediabetic and diabetic disease especially for subtypes of PreDM, may facilitate to find novel strategies which interrupt, reverse, or prevent its initiation prior to clinical disease.

Last but not least, many studies have shown that T2DM can be prevented or delayed by intensive behavioral lifestyle and pharmacological interventions in high-risk populations, especially in subjects with PreDM (10, 11). It is warranted to better identify those at risk and to focus prevention efforts on those who will benefit the most. The OGTT is the gold standard for the diagnosis of PreDM and DM, yet is not popular with primary care physicians and patients. To a large extent, OGTT has been replaced by the more convenient FPG and HbA1c measurements as diagnostic tools (12, 13). However, FPG or HbA1c is prone to miss a considerable number of affected individuals (14). Therefore, developing a simple means for identifying the subjects with PreDM and DM would be very valuable.

In recent decades, the advent of high-resolution and high-sensitivity “omics” techniques has provided clinicians with an additional avenue to monitor disease-related abnormalities from a molecular level perspective (15). Lipidomics can capture both endogenous and exogenous lipidome changes in living systems in response to internal and external perturbations and thus confers further insights into the intricate pathophysiology of diseases (15). A growing number of studies have been focused on the relationship between dysregulation of lipid metabolism and the pathogenesis of T2DM and/or PreDM (16–19). An updated systematic review and meta-analysis of prospective cohort studies identified 62 metabolomics reports testing prospective associations between metabolites/lipids and (pre)diabetes progression (16). The Prevencion con Dieta Mediterranea (PREDIMED) study reported that the baseline levels of glycerides and phosphatidylethanolamines (PE) exhibited highly positive associations with the development of T2DM (18). Some recent studies have also shown that glycerophospholipids (GPL), fatty acids (FA) and acylcarnitines are also associated with the onset and development of T2DM (19–21). These results demonstrated that the lipidomics data can provide important information about diabetes and its progression. However, it is also evident that the identification and screening of prediabetic population, especially for subtypes of PreDM were neglected.

In this study, we conducted a pseudotageted lipidomics analysis for 959 serum samples from multiple communities in Shanghai, China, including 469 newly diagnosed T2DM patients, 301 subjects of three subtypes of PreDM and 189 individuals with normal glucose tolerance. Our aims were to 1) explore distinct differences in serum lipidome from the aspect of the molecular level during the progression of prediabetic and diabetic individuals beyond the well-known differences in dyslipidemia in clinic; 2) identify specific lipid (sub)species associated with each subtype of PreDM; and 3) define diagnostic lipid markers, which is suitable for improving the efficiency and accuracy of current routine (pre)diabetic screening.

## Materials and methods

### Participants

A total of 959 human fasting serum samples for pseudotargeted lipidomics analyses were collected using Vacuette gel plastic tubes from Shanghai Jiao Tong University Affiliated Sixth People’s Hospital (Shanghai, China). All of the serum samples were stored at -80°C prior to sample pre-treatment.

Diagnostic criteria were applied according to the standards of medical care for T2DM in China 2019 (22), which adopts the World Health Organization (WHO 1999) diagnostic criteria. PreDM is divided into three different subcategories: impaired glucose tolerance (IGT) with 7.8 mmol/L ≤ 2h-PG< 11.1, impaired fasting glucose (IFG) with 6.1 mmol/L ≤ FPG< 7.0 and IGT&IFG with 7.8 mmol/L ≤ 2h-PG< 11.1 and 6.1 mmol/L ≤ FPG< 7.1 based on an Oral glucose tolerance test (OGTT) data. The study was approved by the Ethical Committees of Shanghai Jiao Tong University Affiliated Sixth People’s Hospital and performed according to the declaration of Helsinki of 1964 and its later amendments. All participants provided informed written consents.

### Materials and chemicals

HPLC grade acetonitrile (ACN), methanol (MeOH) and isopropanol (IPA) were purchased from Merck (Darmstadt, Germany). HPLC grade methylene chloride (CH_2_Cl_2_), tert-butyl methyl ether (MTBE) and ammonium acetate (AmAc) were purchased from Sigma-Aldrich (St. Louis, MO, USA). Ultrapure water was obtained by the Milli-Q system (Millipore, Billerica, MA). Lipid standards including phosphatidylcholine (PC) 19:0/19:0, lysoPC (LPC) 19:0, PE 17:0/17:0 and PE 15:0/15:0, phosphatidylserine (PS) 17:0/17:0, phosphatidylglycerol (PG) 17:0/17:0, sphingomyelin (SM) d18:1/12:0, ceramide (Cer) d18:1/17:0, diacylglycerol (DG) 12:0/12:0 and DG 14:0/14:0, triacylglycerol (TG) 15:0/15:0/15:0 and TG 20:0_20:1_20:0-d5, FA 16:0_d3 and FA 18:0_d3 were purchased from Avanti polar lipid (Alabaster, AL).

### Sample preparation

Briefly, 150 μL of cold MeOH containing FA 16:0_d3 and FA 18:0_d3 at 0.67 µg/mL, LPC 19:0 at 0.33 µg/mL, PC 19:0/19:0 at 0.67 µg/mL, PE 17:0/17:0 and PE 15:0/15:0 at 0.33 µg/mL, PS 17:0/17:0 at 0.35 µg/mL, PG 17:0/17:0 at 0.35 µg/mL, SM d18:1/12:0 at 0.17 µg/mL, DG 12:0_12:0 and DG 14:0_14:0 at 0.5 µg/mL, TG 15:0/15:0/15:0 and TG 20:0_20:1_20:0-d5 at 0.53 µg/mL was added to 20 μL of each serum or quality control (QC) sample followed by the addition of 500 μL of MTBE. After that, the mixture was vortexed for 10 min. And then 150 μL of ultrapure water was added to the mixture to form a two-phase system. Subsequently, the mixture was vortexed for 60 s and then centrifuged at 13,000 *g* and 4 °C for 10 min. In the end, 200 µL of the supernatant was lyophilized and stored at -80 °C prior to LC-MS analysis. The lyophilized residues were resuspended in ACN/IPA/H2O (65:30:5, v/v/v) containing 5 mM of AmAc.

QC sample was prepared by mixing an equal amount of serum from each sample to monitor the stability of the lipidomics analysis process. The serum samples in the discovery and validation sets were randomly analyzed and one blank sample plus one QC sample were inserted in the analytical sequence after every run of 10 serum samples.

### Pseudotargeted lipidomics analysis

ACQUITY UPLC (Waters, Milford, MA, U.S.A.) coupled to a hybrid QqQ-Trap 5500 system (AB SCIEX/MDS-Sciex, Concord, ON, Canad) that equipped with a Turbo ion spray source was used for pseudotargeted lipidomics profiling analysis in the scheduled MRM mode.

Lipid separation was performed on a Waters BEH C8 column (2.1 mm×100 mm, 1.7 µm). The mobile phase consisted of 60:40 (v/v) ACN/H_2_O with 10 mM of AmAc (phase A) and 90:10 (v/v) IPA/ACN containing 10 mM of AmAc (phase B). The flow rate was set at 0.3 mL/min and the column temperature was set at 60°C. The elution gradient was 50% B at 0-1.5 min and increased linearly to 85% B at 9.0 min, and then reached 100% B at 9.1 min, hold for 1.9 min. Finally, the elution gradient was returned to 50% B in 0.1 min, and held for 1.9 min for column equilibrium. The total running time was 13 min.

The MS detection was operated in positive and negative ion modes, respectively. In positive mode, the IonSpray voltage was 5500 V, ion source temperature was set to 500°C, gas 1 (GS1) and gas 2 (GS2) were both set to 50 psi. In negative ion mode, the IonSpray voltage was -4500 V, ion source temperature was set to 550°C, GS1 and GS2 were both set to 40 psi. In both ion modes, the collision gas and the curtain gas were set to “High” and 35 psi, respectively. Lipid ion pairs existing in the serum sample were identified according to the strategy described previously (23).

Considering the large sample size involved in this study, the retention time (*t*_R_) of the detected lipids was corrected from 20 min (24) to 13 min elution gradient by the spiked lipid internal standards to improve the analytical throughput of pseudotargeted lipidomics in the subsequent serum sample analysis. The information of the internal standards is listed in **Supplementary Table S1**.

### Data processing and statistical analysis

The lipidome data collected by the pseudotargeted lipidomics method were processed using MultiQuant software (version 3.0.3, AB SCIEX, Framingham, U.S.A.). The intensities of lipids in each sample were normalized to those of the corresponding lipid internal standards before statistical analysis.

Partial least squares difference analysis (PLS-DA) was performed by SIMCA-P software (Umetrics, Umeå, Sweden). Variable importance in the projection (VIP) generated from PLS-DA model was used for defining lipids that contribute to the classification between groups. Nonparametric tests for individual lipids were performed using the open-source software MultiExperiment Viewer (MeV, version 4.9.0, Dana-Farber Cancer Institute, MA) in Wilcoxon, Mann-Whitney test mode with the significant level of *p*< 0.05 and false discovery rate (FDR)< 0.05. Non-parametric test for total lipid content of each lipid (sub)species was performed using the Statistical Package for the Social Sciences (SPSS, version 19.0, SPSS Inc., USA) and the significant level of was set at *p<* 0.05. Linear regression tests were carried out to test for statistical associations of lipid (sub)species levels with each of subtype of PreDM and T2DM, taking age, sex and BMI as covariates. Binary logistic regression was used to build the model based on the potential biomarkers. A receiver-operating characteristic curve (ROC) was used to evaluate the results of the regression analysis.

## Results

**Figure 1:**
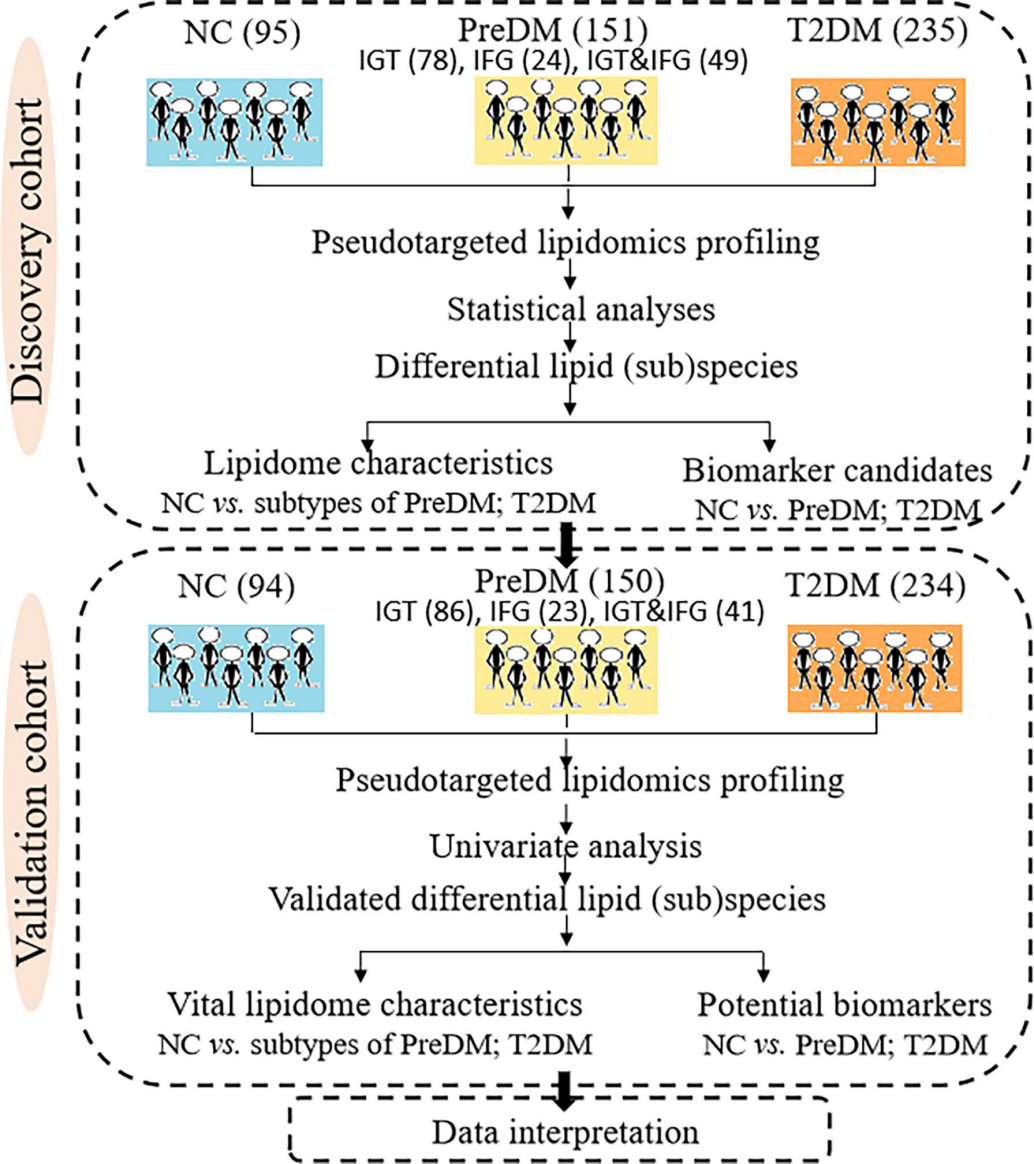
Systematic framework of this study.

The present work applied a two-step analysis strategy including the discovery and validation steps. **Figure 1** shows the overall workflow of the study. A total of 481 participants including 95 NC, 151 PreDM (78 IGT, 24 IFG, 49 IGT&IFG) and 235 T2DM were taken as pseudotargeted lipidomics discovery cohort, while 478 were as the independent external validation cohort containing 94 NC, 150 PreDM (86 IGT, 23 IFG, 41 IGT&IFG) and 234 T2DM.

### Clinical characteristics of the studied subjects

**Table 1** summarizes the clinical characteristics of the studied subjects in the discovery and validation sets in details. It was found that the levels of several clinical characteristics related to diabetes including age, BMI, HOMA-IR, Insulin, FPG, OGTT-0.5h PG, OGTT-1h PG, OGTT-2h PG, OGTT-3h PG and HbA1c were significantly increased in both PreDM and T2DM groups as compared with those in NC group.

**Table 1:** Clinical characteristics of the studied subjects in the discovery and validation sets*.

	Discovery Set			Validation Set		
	NC	PreDM	T2DM	NC	PreDM	T2DM
Sex (male/female)	40/55	53/98	115/120^bb^	36/58	57/93	119/115^ab^
Age (years)	38.5 ± 16.0	51.1 ± 14.6^aaa^	55.9 ± 11.4^aaabb^	38.8 ± 14.2	53.3 ± 12.8^aaa^	55.3 ± 12.3^aaa^
BMI (kg/cm^2^)	24.0 ± 4.6	24.5 ± 4.2	25.1 ± 3.4^aab^	23.6 ± 4.3	24.3 ± 3.8	24.8 ± 3.4^aa^
HOMA - β (%)	134.7 ± 126.7	99.4 ± 119.5^aaa^	68.2 ± 45.5^aaabb^	134.9 ± 101.8	95.1 ± 79.2^aaa^	71.8 ± 53.5^aaabbb^
HOMA - IR	2.3 ± 1.4	2.8 ± 2.5	4.3 ± 2.8^aaabbb^	2.3 ± 1.5	2.7 ± 1.5^a^	4.2 ± 3.0^aaabbb^
FPG (mmol/L)	5.1 ± 0.7	5.9 ± 0.7^aaa^	7.7 ± 1.6^aaabbb^	5.2 ± 0.5	5.9 ± 0.7^aaa^	7.4 ± 1.7^aaabbb^
Insulin (U/L)	10.2 ± 6.0	10.5 ± 9.0	12.8 ± 7.8^aabbb^	10.0 ± 6.2	10.3 ± 5.9	12.6 ± 8.3^ab^
0.5h-PG (mmol/L)	8.5 ± 1.5	10.5 ± 1.4^aaa^	12.8 ± 2.4^aaabbb^	8.6 ± 1.4	10.3 ± 1.6^aaa^	12.3 ± 2.3^aaabbb^
1h-PG (mmol/L)	8.4 ± 2.2	11.6 ± 2.1^aaa^	15.8 ± 3.3^aaabbb^	8.3 ± 1.9	11.5 ± 2.2^aaa^	15.5 ± 3.2^aaabbb^
2h-PG (mmol/L)	6.0 ± 1.1	8.9 ± 1.4^aaa^	15.3 ± 4.0^aaabbb^	6.1 ± 1.1	8.8 ± 1.4^aaa^	14.6 ± 4.0^aaabbb^
3h-PG (mmol/L)	4.6 ± 1.3	5.9 ± 1.9^aaa^	10.6 ± 4.3^aaabbb^	4.5 ± 1.3	5.7 ± 1.9^aaa^	10.1 ± 4.1^aaabbb^
HbA1c (%)	5.4 ± 0.4	5.8 ± 0.5^aaa^	6.8 ± 1.1^aaabbb^	5.4 ± 0.5	5.8 ± 0.4^aaa^	6.7 ± 1.0^aaabbb^
SBP (mmHg)	123.0 ± 14.5	131.9 ± 18.4^aaa^	134.2 ± 15.6^aaabbb^	120.5 ± 17.4	128.8 ± 17.2^aa^	134.9 ± 18.4^aaabb^
DBP (mmHg)	73.6 ± 10.2	79.5 ± 11.1^aaa^	80.8 ± 9.4^aaa^	75.1 ± 11.4	77.4 ± 9.1^a^	82.8 ± 11.1^aaabbb^

*: T2DM, PreDM vs. NC, a, p< 0.05; aa, p< 0.01; aaa, p< 0.001. T2DM vs. PreDM, b, p< 0.05; bb p< 0.01; bbb, p< 0.001. Data represent mean ± SD.

### Serum lipidome profiling of PreDM and T2DM

A total of 804 lipids were identified in 20 μL of the serum QC sample, covering common 18 lipid (sub)species including FA, LPC, PC, PE, DG, TG, etc. Typical chromatograms of lipids detected in QC samples are shown in **Supplementary Figures S1A, B** in positive and negative ion modes, respectively. To assess the data quality throughout the analysis, the relative standard deviation (RSD) of the normalized lipidome data was calculated for all QC samples (**Supplementary Figures S1C, D**). It was observed that RSDs of 76% and 73% of lipids were less than 20% and RSDs of 89% and 88% of lipids were less than 30% in the discovery and validation sets, respectively. Only lipids with RSDs less than 20% in QC samples were included for the subsequent statistical data analysis.

First of all, orthogonal signal correction-PLS-DA (OSC-PLS-DA) was performed to obtain the overall lipidomic profile differences in PreDM versus NC and T2DM versus NC. In the discovery set, both PreDM and T2DM groups were clearly separated from the NC group (**Supplementary Figures S2A, B**), implying that the lipid molecular characteristics of PreDM and T2DM were different from those of NC. In addition, there was also a clear trend of separation between PreDM and T2DM (**Supplementary Figure S2C**), indicating that the lipidomic pattern of T2DM was different from that of PreDM. Similar findings were also obtained in the validation set (**Supplementary Figures S2D-F**).

### Differences in lipid (sub)species for PreDM subtypes and T2DM

**Figure 2:**
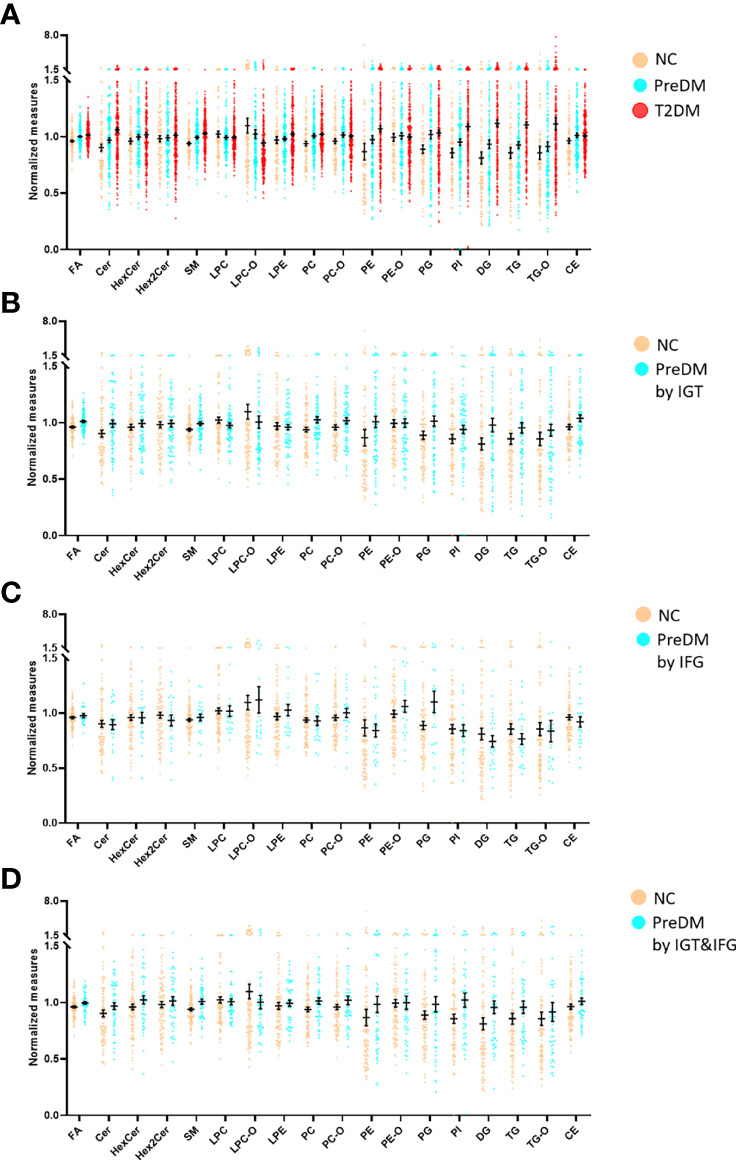
Mean normalized lipid (sub)species levels in T2DM, PreDM and NC by **(A)** any of the three criteria for PreDM, **(B)** IGT, **(C)** IFG and **(D)** IGT&IFG in the discovery set. Data are expressed as means ± SEM.

Differences at 18 lipid (sub)species levels were further investigated among individuals with PreDM, T2DM and those with normoglycemia in the discovery (**Figure 2**) and validation sets (**Supplementary Figure S3**). The results showed distinctive lipidome signatures for NC, each subtype of PreDM and T2DM individuals. The statistical significance of differences with *p* value< 0.05 in lipid (sub)species distributions among normoglycemic, prediabetic and diabetic individuals were determined using non-parametric test (Mann-Whitney test) for both sets (**Table 2**). Significant increase in FA, Cer, SM, lysoPE (LPE), PC, PE, phosphatidylinositol (PI), DG, TG, TG with alkyl/alkenyl ether substituents (TG-O) and cholesterol ester (CE) levels were found in patients with T2DM. FA, Cer, SM, PC, PC with alkyl/alkenyl ether substituents (PC-O), PE, DG and TG levels were differentially distributed among individuals with PreDM and individuals with normoglycaemia. Among the subtypes of PreDM, SM, PC, PE, DG and TG were associated for PreDM defined by IGT and IGT&IFG, while FA and Cer were associated with prediabetic status defined by IGT&IFG. Only PG level was identified to be significantly associated with PreDM defined by IFG. After statistical differences in lipid (sub)species levels by linear regression with sex, age and BMI as covariates (**Table 3**), FA, Cer, SM, LPC with alkyl/alkenyl ether substituents (LPC-O), PC, PE, PI, DG and TG showed a significant association with T2DM, while FA and PC were associated at *p*< 0.05 for all PreDM. In the subtypes of PreDM, only PC significantly associated with prediabetic status defined by IGT and IGT&IFG, and no lipid (sub)species show significant changes for PreDM defined by IFG.

**Table 2:** Statistical differences in lipid (sub)species levels by Mann-Whitney test.

Lipid (sub)species	Discovery set					Validation set				
	PreDM (All)	PreDM by IGT	PreDM by IFG	PreDM by IGT&IFG	T2DM	PreDM (All)	PreDM by IGT	PreDM by IFG	PreDM by IGT&IFG	T2DM
	n = 151	n = 78	n = 24	n = 49	n = 235	n = 150	n = 86	n = 23	n = 41	n = 234
	*p*	*p*	*p*	*p*	*p*	*p*	*p*	*p*	*p*	*p*
FA	0.003	0.001	0.776	< 0.001	< 0.001	0.017	0.069	0.442	0.011	< 0.001
Cer	0.007	0.011	0.483	0.024	< 0.001	0.023	0.147	0.891	0.001	< 0.001
HexCer	0.261	0.538	0.848	0.110	0.136	0.809	0.610	0.384	0.402	0.793
Hex2Cer	0.845	0.860	0.583	0.579	0.463	0.997	0.868	0.794	0.916	0.869
SM	0.001	0.005	0.252	0.005	< 0.001	0.001	0.049	0.022	< 0.001	< 0.001
LPC	0.941	0.767	0.801	0.675	0.721	0.621	0.973	0.593	0.089	0.695
LPC-O	0.468	0.942	0.357	0.329	0.989	0.425	0.538	0.671	0.188	0.452
LPE	0.151	0.470	0.199	0.142	0.022	0.084	0.244	0.696	0.027	0.008
PC	0.019	0.018	0.837	0.026	< 0.001	< 0.001	< 0.001	0.092	0.001	< 0.001
PC-O	0.029	0.04	0.151	0.208	0.016	0.029	0.203	0.062	0.030	0.058
PE	< 0.001	< 0.001	0.216	0.018	< 0.001	0.001	0.003	0.451	0.003	< 0.001
PE-O	0.765	0.954	0.162	0.776	0.683	0.544	0.132	0.595	0.552	0.86
PG	0.016	0.028	0.023	0.305	< 0.001	0.327	0.817	0.032	0.160	0.064
PI	0.005	0.006	0.868	0.009	< 0.001	0.416	0.171	0.524	0.518	0.019
DG	0.003	0.009	0.751	0.003	< 0.001	0.013	0.006	0.131	0.004	< 0.001
TG	0.016	0.017	0.817	0.018	< 0.001	0.023	0.010	0.188	0.015	< 0.001
TG-O	0.121	0.077	0.791	0.344	< 0.001	0.294	0.542	0.484	0.031	0.001
CE	0.110	0.064	0.404	0.103	0.019	< 0.001	0.010	0.079	< 0.001	0.002

**Table 3:** Statistical differences in lipid (sub)species levels by linear regression, with sex, age and BMI as covariates.

Lipid (sub)species	Discovery set										Validation set									
	PreDM (All)		PreDM by IGT		PreDM by IFG		PreDM by IGT&IFG		T2DM		PreDM (All)		PreDM by IGT		PreDM by IFG		PreDM by IGT&IFG		T2DM	
	n = 151		n = 78		n = 24		n = 49		n = 235		n = 150		n = 86		n = 23		n = 41		n = 234	
	β	*P*	β	*P*	β	*P*	β	*P*	β	*P*	β	*P*	β	*P*	β	*P*	β	*P*	β	*P*
FA	0.369	0.018	0.515	0.006	0.172	0.480	0.391	0.059	0.617	< 0.001	0.321	0.042	0.286	0.118	0.167	0.543	0.539	0.023	0.714	< 0.001
Cer	0.272	0.063	0.356	0.039	-0.008	0.973	0.300	0.121	0.615	< 0.001	0.248	0.119	0.231	0.188	-0.118	0.691	0.531	0.024	0.628	< 0.001
HexCer	0.125	0.395	0.084	0.628	-0.042	0.860	0.302	0.128	0.149	0.335	-0.054	0.725	-0.020	0.912	-0.218	0.423	0.161	0.462	-0.065	0.652
Hex2Cer	0.073	0.614	0.087	0.610	-0.135	0.579	0.213	0.273	0.210	0.163	0.042	0.782	0.103	0.564	0.110	0.661	0.055	0.801	0.026	0.862
SM	0.283	0.060	0.251	0.138	0.047	0.838	0.334	0.086	0.513	0.001	0.554	0.001	0.338	0.067	0.578	0.034	0.826	0.001	0.477	0.003
LPC	-0.239	0.097	-0.350	0.054	-0.233	0.363	-0.247	0.228	-0.384	0.007	0.064	0.681	-0.101	0.554	0.015	0.953	0.117	0.582	-0.239	0.099
LPC-O	-0.240	0.095	-0.328	0.070	-0.154	0.548	-0.376	0.087	-0.518	< 0.001	0.049	0.764	-0.067	0.695	-0.011	0.963	-0.014	0.946	-0.374	0.006
LPE	-0.076	0.599	-0.204	0.255	0.065	0.788	-0.018	0.929	-0.013	0.933	0.119	0.462	0.020	0.909	0.098	0.699	0.167	0.434	0.228	0.144
PC	0.347	0.026	0.408	0.023	-0.024	0.920	0.392	0.050	0.385	0.014	0.571	0.001	0.555	0.004	0.574	0.038	0.634	0.007	0.586	0.001
PC-O	0.287	0.062	0.307	0.078	0.292	0.208	0.285	0.131	0.282	0.054	0.359	0.023	0.303	0.096	0.635	0.021	0.502	0.030	0.275	0.065
PE	0.162	0.291	0.202	0.145	-0.081	0.777	0.174	0.338	0.367	0.037	0.328	0.054	0.292	0.121	0.026	0.931	0.522	0.018	0.836	< 0.001
PE-O	0.066	0.645	0.056	0.738	0.312	0.179	0.066	0.725	0.206	0.164	-0.019	0.904	-0.150	0.433	0.236	0.415	0.232	0.321	0.090	0.552
PG	0.334	0.036	0.331	0.057	0.453	0.037	0.211	0.250	0.523	0.002	0.169	0.287	0.098	0.580	0.450	0.100	0.315	0.171	0.243	0.126
PI	0.220	0.156	0.152	0.368	-0.123	0.625	0.388	0.052	0.579	0.001	-0.001	0.997	-0.085	0.623	-0.015	0.955	0.207	0.336	0.318	0.045
DG	0.286	0.053	0.378	0.028	-0.329	0.312	0.303	0.117	0.526	0.003	0.122	0.457	0.196	0.286	-0.818	0.058	0.297	0.192	0.533	0.003
TG	0.190	0.188	0.266	0.115	-0.361	0.279	0.270	0.155	0.487	0.003	0.137	0.389	0.217	0.235	-0.642	0.110	0.291	0.190	0.508	0.004
TG-O	0.113	0.426	0.200	0.220	-0.033	0.895	0.156	0.397	0.491	0.021	0.007	0.962	0.023	0.896	-0.459	0.244	0.204	0.349	0.313	0.109
CE	0.209	0.148	0.299	0.078	-0.207	0.420	0.158	0.411	0.177	0.219	0.412	0.014	0.350	0.053	0.297	0.276	0.615	0.009	0.300	0.062

### Potential lipid markers for PreDM and T2DM screening

**Figure 3:**
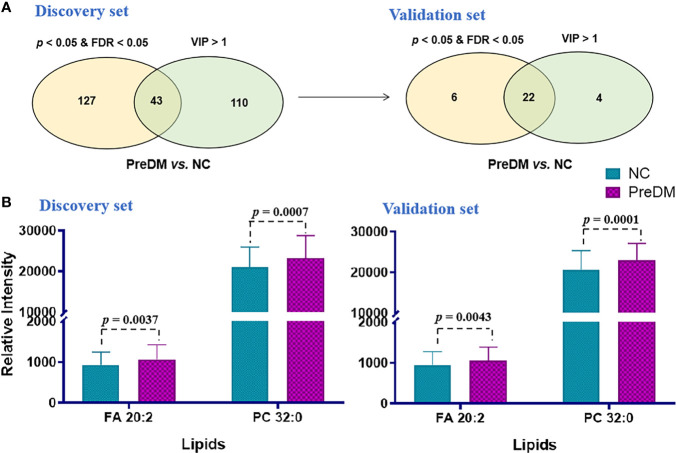
**(A)** Venn diagram of significantly differential lipids based on NC and PreDM. **(B)** Histograms of potential lipid markers in NC and PreDM specimens. Data are presented as mean ± SD.

A PLS-DA model was established to find out the vital variables to distinguish the PreDM from NC groups in the discovery set. A total of 153 lipids with VIP > 1.0 were selected for subsequent univariate analysis to determine whether they were significantly altered in the PreDM group versus the NC group (**Figure 3A**). In total, 43 lipids exhibited *p*< 0.05 and FDP< 0.05 (**Figure 3A**) and were regarded as the lipid biomarker candidates. To define the potential diagnostic lipid biomarkers for PreDM, an independent validation cohort of 478 individuals was used to evaluate the reliability of these 43 biomarker candidates with the criteria of *p*< 0.05, FDR<0.05 and VIP >1. Ultimately, 22 lipids were validated to be potential biomarkers for PreDM (**Figure 3A**). To identify lipid predictors, odds ratios (ORs) of developing PreDM per SD increase in these 22 lipid species were further calculated by conditional logistic regression models after adjusting for age, sex, BMI in the discovery and validation sets, respectively. Two lipids (FA 20:2, PC 32:0) showed significant associations with incident PreDM (**Supplementary Table S2**). Subsequently, a binary logistic regression analysis and an algorithm of the forward stepwise method were employed to construct the optimal model using these 2 potential lipid biomarkers. Finally, FA 20:2 and PC 32:0 were defined as an ideal biomarker panel 1 to distinguish patients with PreDM from NC subjects (**Figure 3B**).

**Figure 4:**
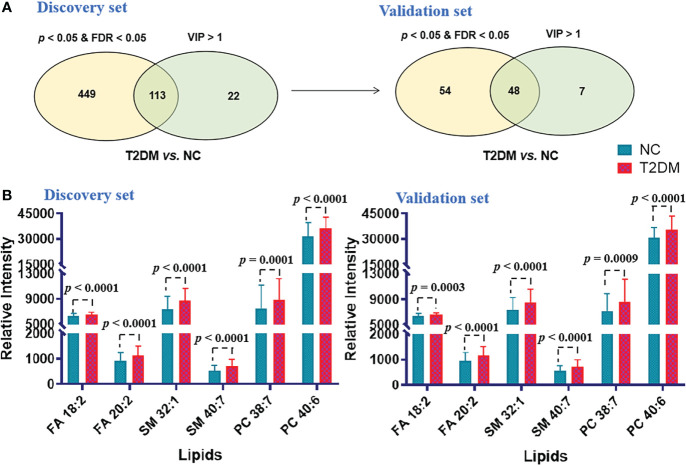
**(A)** Venn diagram of significantly differential lipids based on NC and T2DM. **(B)** Histograms of potential lipid markers in NC and T2DM specimens. Data are presented as mean ± SD.

Similar analytical procedures mentioned above were carried out to identify the ideal biomarker panel to distinguish patients with T2DM from NC subjects. As shown in **Figure 4A**, 48 lipids were defined to be biomarker candidates with *p*< 0.05, FDR< 0.05 and VIP > 1 in the both cohorts. Subsequently, ORs of developing T2DM per SD increase in these 48 lipid species were further calculated by conditional logistic regression models after adjusting for age, sex and BMI in the discovery and validation sets, respectively. Thirty-one lipids showed significant associations with incident T2DM (**Supplementary Table S3**). Additionally, a binary logistic regression analysis and an algorithm of the forward stepwise method were performed to build the optimal model using these 31 potential lipid biomarkers. In the end, lipid molecules of FA 18:2, FA 20:2, SM 32:1, SM 40:7, PC 38:7 and PC 40:6 were selected as the biomarker panel 2 (**Figure 4B**) to distinguish patients with T2DM from NC subjects.

### Diagnostic power of potential lipid markers for PreDM and T2DM

A binary logistic regression model was conducted to test the diagnostic power of potential lipid biomarkers for PreDM and T2DM. ROCs were plotted and the area under receiver-operating characteristic curves (AUC) were obtained to evaluate the accuracy of the diagnostic model based on lipid biomarker candidates and/or clinical characteristics. The diagnostic power of the panel 1 combined with FPG was higher than that of FPG alone in discriminating PreDM from NC in the discovery and validation sets (AUC = 0.850 vs. 0.821 and 0.810 vs. 0.797), respectively (**Table 4**). Furthermore, the diagnostic accuracy of panel 1 combined with FPG (69.5% and 66%) was much higher than FPG alone (48.3% and 42.7%) in identifying patients with PreDM from NC in the discovery and validation sets. Notably, the combination of lipid panel 1 with age, sex, BMI and FPG exhibited more improvement in diagnosing PreDM in the discovery and validation sets (diagnostic accuracy = 77.3% and 73.2%), respectively (**Table 4**).

**Table 4:** AUC and accuracy for diagnosis of PreDM and T2DM in the discovery and validation sets*.

	WHO criteria (6.1 ≤ FPG< 7.0 mmol/L)				ADA criteria (5.6 ≤ FPG< 6.9 mmol/L)			
	Discovery cohort		Validation cohort		Discovery cohort		Validation cohort	
	AUC (95% Cl)	Accuracy	AUC (95% Cl)	Accuracy	AUC (95% Cl)	Accuracy	AUC (95% Cl)	Accuracy
Model for diagnosis of PreDM								
FPG	0.821 (0.770-0.871)	48.3%	0.797 (0.743-0.852)	42.7%	0.901 (0.865-0.938)	73.4%	0.901 (0.863-0.938)	71.7%
FPG/Panel 1	0.850 (0.804-0.897)	69.5%	0.810 (0.756-0.863)	66.0%	0.907 (0.872-0.943)	76.9%	0.905 (0.869-0.941)	76.3%
Age/Sex/BMI/FPG/Panel 1	0.877 (0.834-0.919)	77.3%	0.866 (0.821-0.911)	73.2%	0.910 (0.875-0.945)	78.6%	0.923 (0.891-0.955)	71.7%
Model for diagnosis of T2DM								
FPG	0.982 (0.971-0.993)	68.1%	0.951 (0.929-0.973)	59.0%	0.993 (0.987-1.000)	68.1%	0.975 (0.958-0.992)	59.0%
FPG/Panel 2	0.986 (0.977-0.995)	92.3%	0.964 (0.945-0.983)	88.9%	0.995 (0.989-1.000)	97.4%	0.978 (0.963-0.993)	94.4%
Age/Sex/BMI/FPG/Panel 2	0.992 (0.986-0.998)	93.2%	0.975 (0.960-0.991)	94.0%	0.997 (0.993-1.000)	97.0%	0.990 (0.982-0.998)	95.3%

*: Panel 1, FA 20:2, PC 32:0; Panel 2, FA 18:2, FA 20:2, SM 32:1, SM 40:7, PC 38:7 and PC 40:6.

For the diagnosis of T2DM, the panel 2 combined with FPG had a similar AUC to that of FPG (i.e., 0.986 and 0.964 versus 0.982 and 0.951) in the discovery and validation sets, respectively (**Table 4**). However, this serum lipid panel 2 combined with FPG showed better diagnostic accuracy when compared with FPG (92.3% versus 68.1%, and 88.9% versus 59.0% in the discovery and validation sets) in identifying patients with T2DM from NC. Furthermore, the combination of this lipid panel 2 with age, sex, BMI and FPG had an even more accuracy in diagnosing T2DM (accuracy = 93.2% and 94.0% in the discovery and validation sets) (**Table 4**). Additionally, similar results were obtained when using the American Diabetes Association (ADA) criteria which defines IFG as 5.6 to 6.9 mmol/L (**Table 4**).

**Figure 5:**
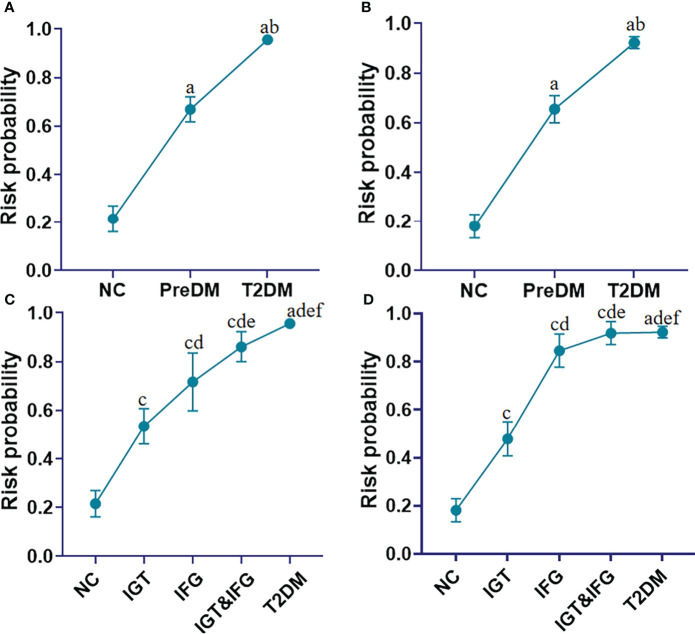
Line charts present the risk probability of developing into T2DM in the cohorts of the NC, PreDM and T2DM in the discovery **(A)** and validation **(B)** sets based on addition of FA 18:2, FA 20:2, SM 32:1, SM 40:7, PC 32:0, PC 38:7, PC 40:6 to Age, BMI, and FPG; Line charts present the risk probability of developing into T2DM in the cohorts of the NC, subgroups of PreDM including IGT to IFG to IFG&IGT, and T2DM in the discovery **(C)** and validation **(D)** sets based on addition of FA 18:2, FA 20:2, SM 32:1, SM 40:7, PC 32:0, PC 38:7, PC 40:6 to Age, BMI, and FPG. ^a^ *P*< 0.05, when T2DM and PreDM are compared with NC; ^b^ *P*< 0.05, when T2DM is compared with PreDM, ^c^ *P*< 0.05, when IGT, IFG and IGT&IFG is compared with NC, ^d^ *P*< 0.05, when IFG, IGT&IFG and T2DM is compared with IGT, ^e^ *P*< 0.05, when IGT&IFG and T2DM is compared with IFG, ^f^ *P*< 0.05, when T2DM is compared with IGT&IFG. Data represent mean with 95% CI.

To assess whether the panel 1 and the panel 2 have the ability to identify the risk of diabetes, the risk probability of developing into diabetes was calculated by an equation constructed from the above two panels of 7 lipid markers (i.e., FA 18:2, FA 20:2, SM 32:1, SM 40:7, PC 32:0, PC 38:7 and PC 40:6), age, BMI and FPG. The constructed equation is displayed as follows:


$$\begin{array}{l} \text{Logit}\;\left[\text{P}=\text{T}2\text{DM}\right]=6.39*10^{-4}\times \left[\text{FA}\;18:2\right]+2.54*10^{-3}\times \left[\text{FA}\;20:2\right]\\ +8.60*10^{-5}\times [\text{SM}\;32:1]+9.09*10^{-4}\times \left[\text{SM}\;40:7\right]+1.26*10^{-5}\\ \times \left[\text{PC}\;32:0\right]+4.32*10^{-5}\times \left[\text{PC}\;38:7\right]+4.08*10^{-5}\times \left[\text{PC}\;40:6\right]+2.47\\ \times \left[\text{FPG}\right]+8.74*10^{-2}\times \left[\text{Age}\right]+9.55\;*\;10^{-2}\times \left[\text{BMI}\right]-30.46 \end{array}$$

where [P = T2DM] is the risk probability of developing into diabetes with this panel, and [FA 18:2], [FA 20:2], [SM 32:1], [SM 40:7], [PC32:0], [PC 38:7] and [PC 40:6] mean the relative concentrations of these lipid molecules. The units of [FPG], [Age], [BMI] are mmol/L, year, kg/cm^2^, respectively. We observed that the risk probability of developing into diabetes gradually increased from the NC, PreDM, ultimately to T2DM in the discovery set (**Figure 5A**). More interestingly, the risk probability of developing into diabetes increases sequentially from impaired glucose tolerance (IGT), impaired fasting glucose (IFG) to IGT&IFG (**Figure 5C**). Similar results were obtained in the validation set (**Figures 5B, D**).

### Associations of the biomarker panels and lipid (sub)species with HOMA-β and HOMA-IR

As the identified biomarker panel consisting of FA 18:2, FA 20:2, SM 32:1, SM 40:7, PC 32:0, PC 38:7 and PC 40:6 was confirmed to have high diagnostic power for PreDM and T2DM in two independent cohorts, it may mean a major association of these lipids with the pathogenesis of T2DM. To reveal the possible mechanisms of the found lipid panels associated with PreDM and T2DM risks, we first performed a Pearson correlation analysis of these 7 lipids with HOMA-β and HOMA-IR — the two common diabetes parameters. The result showed that FA 18:2 and SM 40:7 displayed a significant negative correlation with HOMA-β (*P*< 0.05). To further reveal the biological relevance of lipidomic profiles and insulin sensitivity and/or β-cell function, we have also tested associations between HOMA-β, HOMA-IR and distinctly differential lipid (sub)species (e.g., FA, Cer, SM, PC, PE, DG, and TG) among diabetic, prediabetic and normoglycemic individuals. The outcome revealed that Cer had a significant negative correlation with HOMA-β (*P*< 0.05), while PI, TG-O and DG had a significant positive correlation with HOMA-IR (*P*< 0.05).

## Discussion

T2DM and PreDM is highly associated with metabolic dysregulations, including hyperglycemia and hyperlipemia. Multiple high-risk factors including age, genetic factors, smoking, alcohol consumption, obesity, hyperglycemia, hypertension, hyperlipidemia, etc. leading to diabetes have been identified. However, these factors provide limited information for understanding the metabolic disturbances in T2DM and PreDM. Lipids are essential components of cell membrane structure and key regulators of the cell cycle and physiological processes. A comprehensive lipidomics study on PreDM subtypes and T2DM can provide in-depth insights into the onset and development of diabetes.

Our results show that there are distinct differences in lipidome mainly related to metabolism of FA, Cer, SM, PC, PE, DG, and TG among diabetic, prediabetic and normoglycemic individuals. The degree of disorder in Cer, PE, DG and TG was gradually increasing in the progression of PreDM to T2DM. Adjusted for sex, age and BMI, we found that the mean levels of FA and Cer in T2DM were still much higher than those in normoglycemic individuals. It has been previously reported that lipids involved in the fatty acid and sphingolipid metabolism pathways show different signatures between normoglycemic individuals and those with T2DM (25, 26). We also observed that the mean levels of FA and Cer of individuals with PreDM were noticeably higher than those of NC when adjusted for sex, age and BMI (**Table 3**). Cer is a bioactive sphingolipid that is responsible for signaling transmission and is closely related to β-cell function and insulin sensitivity (27, 28). Consistent with these findings, the level of circulating Cer was significantly and negatively associated with the level of HOMA-β (*P*< 0.05). Interestingly, all of these elevated Cer contain saturated FA chains, showing a gradual upward trend as diabetes progresses (**Table 5**). We speculated that the elevated Cer with saturated FA may be more relevant with high insulin levels and HOMA-IR, exacerbating the development from PreDM to diabetes as compared with other types of Cer. However, the detailed mechanism needs to be further studied.

**Table 5:** Significantly changed ceramides in the T2DM and PreDM *vs.* NC.

Lipids	Discovery set						Validation set					
	PreDM *vs.* NC			T2DM vs NC			PreDM *vs.* NC			T2DM vs NC		
	*p*	FDR	FC	*p*	FDR	FC	*p*	FDR	FC	*p*	FDR	FC
Cer(d18:1/16:0)	3.78E-03	3.27E-02	1.08	2.98E-06	9.07E-06	1.16	4.45E-02	1.14E-01	1.05	3.63E-05	1.85E-04	1.12
Cer(d18:1/18:0)	9.19E-03	4.65E-02	1.14	1.12E-05	2.97E-05	1.22	7.32E-03	3.32E-02	1.15	6.73E-05	2.83E-04	1.20
Cer(d16:1/22:0)	3.78E-02	1.04E-01	1.09	7.43E-09	6.47E-08	1.29	5.91E-03	2.99E-02	1.19	2.70E-06	2.91E-05	1.33
Cer(d18:1/22:0)	2.55E-02	8.11E-02	1.06	7.56E-07	2.78E-06	1.19	1.60E-02	5.61E-02	1.10	9.29E-07	1.44E-05	1.21
Cer(d18:1/23:0)	5.14E-03	3.61E-02	1.09	2.42E-06	7.67E-06	1.18	4.86E-02	1.20E-01	1.09	6.88E-05	2.88E-04	1.19
Cer(d18:1/24:0)	4.66E-03	3.41E-02	1.08	3.93E-07	1.60E-06	1.19	3.13E-02	8.94E-02	1.10	3.43E-06	3.36E-05	1.20

We also showed differences in lipidome between subtypes of PreDM. For example, the mean levels of SM, PC, PE, DG and TG of individuals with prediabetes defined by IGT or IGT&IFG were much higher than those of normoglycemic individuals. And the mean levels of FA and Cer of individuals with prediabetic status defined by IGT&IFG were also higher than those of NC. However, only PG level was identified to be significantly associated with PreDM defined by IFG. When adjusted for sex, age and BMI, the mean PC levels of individuals with PreDM defined by IGT and IGT&IFG were also higher than normal, but the difference was non-significant for IFG defined prediabetes, suggesting that PC levels may interact differently with the metabolic drivers of fasting and post-load glucose levels. PC are well known as the most important components of the phospholipid bilayer of the cell membrane. The imbalance of PC will greatly affect the physicochemical properties of the cell membrane, leading to cellular dysfunction (29). Previous studies have also found that PC metabolism was abnormal in diabetes and its complications, characterized by an elevation of PC (20). We deduced that the significant increase in PC may be preceded by diabetes and its complications.

Globally, the incidence of diabetes and prediabetes increases year by year. Up to date, accurate and highly-efficient diagnosis of T2DM and PreDM is only feasible by applying a 75 g OGTT (30). However, an OGTT is a laborious, time-consuming and error-prone process due to frequent blood sampling over 2 hours, which is not suitable for a large-scale diabetes screening. The analysis of FPG or HbA1c is commonly used for PreDM and T2DM screening, but is prone to miss a considerable number of affected individuals (8, 31). In the current study, we identified and validated two lipid marker panels in distinguishing patients with PreDM and T2DM from NC subjects with high diagnostic power. The combination of the panel 1 with age, sex, BMI and FPG significantly improved the diagnostic accuracy of broad-scale PreDM screening (73.2% versus 42.7%). The combination of the panel 2 with age, sex, BMI and FPG significantly improved the diagnostic accuracy of broad-scale T2DM screening (94.0% versus 59.0%). It led to a striking decrease from > 50% and > 40% missed diagnoses by using FPG down to 16.8% and 6% by applying lipid panel + age + sex + BMI + FPG for PreDM and T2DM in the validation set, respectively. These results demonstrated that the two serum lipid panels had the potential to screen patients with PreDM and T2DM from healthy populations without performing an OGTT in a large-scale population.

Individuals with PreDM already experience a extent of lipidome variations and are likely to develop overt T2DM (4). It needs to better identify those at risk and to focus prevention efforts on those who would benefit most. The combination of panel 1 and panel 2 with age, BMI and FPG enabled to efficiently evaluate the risk of developing into diabetes from each subtype of PreDM. We found that although IGT, IFG as well as IGT&IFG are all subtypes of PreDM, IFG and IGT&IFG are more likely to develop into diabetes than IGT in our study. Collectively, these findings are helpful to raise the awareness of the risk of different subtypes of diabetes, and provide the evidence for early intervention of these lipid markers to reduce the progression from prediabetes to diabetes. However, our cross-sectional study has some limitations, the risk assessment of these identified lipid markers for the development of diabetes requires further validation in prospective cohorts.

Last but not least, all of the 7 lipids included in the panels 1 and 2 showed significant changes in serum levels from normal glucose tolerant individuals to PreDM to manifest T2DM (**Figures 3B**, **4B**). Furthermore, we found that FA 18:2 and SM 40:7 from the panels 1 and 2 showed a significant negative correlation with HOMA-β (*P*< 0.05). We speculated that increased FA 18:2 and SM 40:7 levels may impair beta-cell function rather than insulin sensitivity and thus contribute to (pre)diabetes, but further investigation needs to be conducted in the future.

## Conclusions

In the present study, a high-coverage pseudotargeted lipidomics method was used to uncover distinctive lipidome signatures between groups among normoglycemic, prediabetic and diabetic individuals, including energy metabolism related lipid (sub)species (FA, DG, TG) as previously reported, and distinctive signatures in PC levels between different subtypes of PreDM. The identified lipid markers significantly improved the diagnostic accuracy of PreDM and T2DM when combined with age, sex, BMI, and FPG, not only reducing FPG-false-negative missed detections, but also effectively evaluating the risk of developing into each subtype of PreDM and T2DM. Our findings demonstrated that lipidomics data provide a high-dimensional lipidome changing snapshot beyond the well-known differences in dyslipidemia in clinic in the early developmental stages of T2DM and improve insights to lipid metabolic complexity and interindividual variations in PreDM and T2DM.

## Data availability statement

The original contributions presented in the study are included in the article/**Supplementary Material**. Further inquiries can be directed to the corresponding authors.

## Ethics statement

The studies involving human participants were reviewed and approved by Ethics Committee of Shanghai Sixth People’s Hospital. The patients/participants provided their written informed consent to participate in this study.

## Author contributions

GX, WJ, and CW conceptualized and designed the study, and reviewed and revised the manuscript. QX performed the initial analyses, and drafted the initial manuscript. CH collected the data, and reviewed and revised the manuscript. YZ collected the samples. QW, XZ, and XL reviewed and revised the manuscript. GX, WJ, CW and QX are the guarantors of this work and, as such, have full access to all the data in the study and take responsibility for the integrity of the data and the accuracy of the data analysis. All authors contributed to the article and approved the submitted version.

## Funding

This research was supported by the mobility programme of the Sino-German Center for Research Promotion (M-0257), the key foundation (2019J11CY018) of Dalian City, the National Natural Science Foundation of China (No. 21874130), and the Innovation Program (DICP ZZBS201804, DICP I202019) of Science and Research from the DICP, CAS.

## Conflict of interest

The authors declare that the research was conducted in the absence of any commercial or financial relationships that could be construed as a potential conflict of interest.

## Publisher’s note

All claims expressed in this article are solely those of the authors and do not necessarily represent those of their affiliated organizations, or those of the publisher, the editors and the reviewers. Any product that may be evaluated in this article, or claim that may be made by its manufacturer, is not guaranteed or endorsed by the publisher.
