# Supplementary material for: Serum lipidomics profiles reveal potential lipid markers for prediabetes and type 2 diabetes in patients from multiple communities
Source: Front Endocrinol (Lausanne). 2022 Aug 15;13:966823. doi: 10.3389/fendo.2022.966823 (PMC9434798; doi:10.3389/fendo.2022.966823)
Supplement: Supplementary file 1 [file DataSheet_1.docx]

Serum Lipidomics Profiles Reveal Potential Lipid Markers for Prediabetes and Type 2 Diabetes in Patients from Multiple Communities

Qiuhui Xuan^1,2,3^, Chunxiu Hu^1,3^, Yinan Zhang^4^, Qingqing Wang^1,3^, Xinjie Zhao^1,3^, Xinyu Liu^1,3^, Congrong Wang^4,5*^, Weiping Jia^4*^, Guowang Xu^1,3*^

^1^ CAS Key Laboratory of Separation Sciences for Analytical Chemistry, Dalian Institute of Chemical Physics, Chinese Academy of Sciences, 457 Zhongshan Road, Dalian, 116023, China

^2^ Department of Endocrinology, Shandong Provincial Hospital, Shandong University, Jinan, Shandong, China

^3^ University of Chinese Academy of Sciences, Beijing, 100049, China

^4^ Shanghai Diabetes Institute, Shanghai Key Laboratory of Diabetes Mellitus, Shanghai Clinical Center for Endocrine and Metabolic Diseases, Metabolic Diseases Biobank, Shanghai Jiao Tong University Affiliated Sixth People's Hospital, Shanghai 200233, China

^5^ Shanghai Fourth People’s Hospital, Department of Endocrinology and Metabolism, Tongji University School of Medicine, Shanghai 200434, China

*: Corresponding authors to whom reprint requests should be addressed:

Prof. Dr. Guowang Xu, CAS Key Laboratory of Separation Science for Analytical Chemistry, Dalian Institute of Chemical Physics, Chinese Academy of Sciences, Dalian 116023, China. orcid.org/0000-0003-4298-3554; Tel. / Fax: 0086-411-84379530. E-mail: [xugw@dicp.ac.cn](mailto:xugw@dicp.ac.cn)

Prof. Dr. Weiping Jia, Shanghai Diabetes Institute, Shanghai Key Laboratory of Diabetes Mellitus, Shanghai Clinical Center for Endocrine and Metabolic Diseases, Metabolic Diseases Biobank, Shanghai Jiao Tong University Affiliated Sixth People’s Hospital, Shanghai 200233, China. Email: wpjia@sjtu.edu.cn

Prof. Dr. Congrong Wang, Shanghai Diabetes Institute, Shanghai Key Laboratory of Diabetes Mellitus, Shanghai Clinical Center for Endocrine and Metabolic Diseases, Metabolic Diseases Biobank, Shanghai Jiao Tong University Affiliated Sixth People’s Hospital, Shanghai 200233, China; Shanghai Fourth People’s Hospital, Department of Endocrinology and Metabolism, Tongji University School of Medicine, Shanghai 200434, China; Email: crwang@tongji.edu.cn

Supplementary materials

Figure S1. Typical chromatograms of serum sample in positive and negative ion modes…………………………………………………………………………… ... S4

Figure S2. Score plots of orthogonal signal correction-partial least square discriminant analysis for NC, PreDM and T2DM……………………………………………… ..S5

Figure S3. Mean normalised lipid (sub)species levels in T2DM, PreDM and NC by (a) any of the three criteria for PreDM, (b) IGT, (c) IFG and (d) IGT&IFG in the validation set.……………………………………………… …………………………………..S6

Table S1. Retention time and ion pair information of lipid internal standards….. …S7

Table S2. Key lipids: ORs of PreDM versus NC after adjusting for age, sex and BMI…..... ..... ..... ..... ..... ..... ..... ..... ..... ..... ..... ..... ..... ... ... ... ... ... ... ... ... ....... S8

Table S3. Key lipids: ORs of T2DM versus NC after adjusting for age, sex and BMI….. ..... ..... ..... ..... ..... ..... ..... ..... ..... ..... ..... ..... ... ... ... ... ... ... ... ... ... ... ..S9-S10


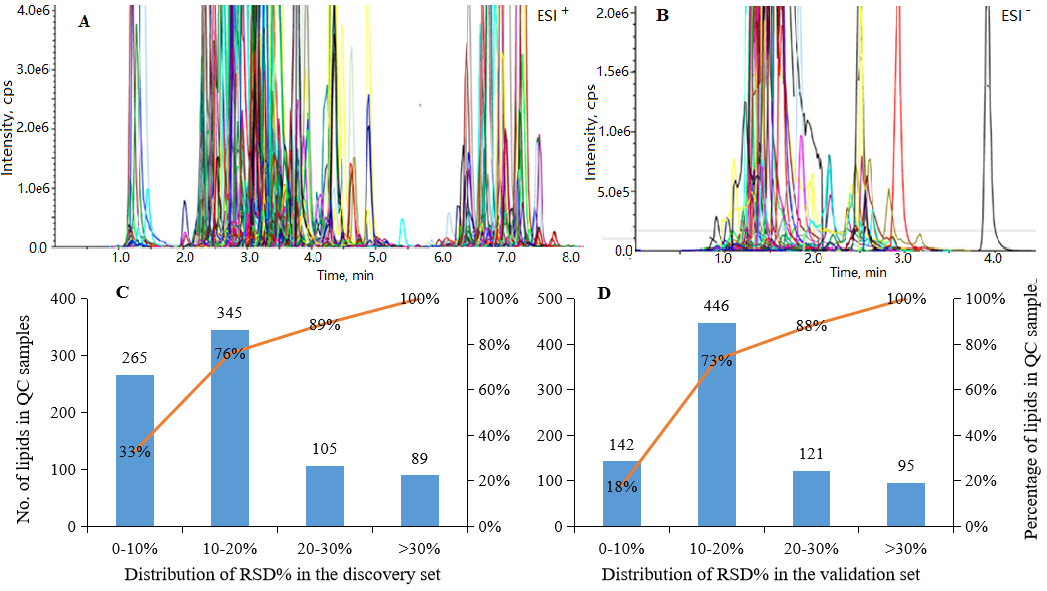


Figure S1. Typical chromatograms of serum sample in positive (A) and negative (B) ion modes, respectively. Evaluation of data quality for pooled QC samples inserted in analysis batch per 10 samples in the discovery (C) and validation (D) sets.


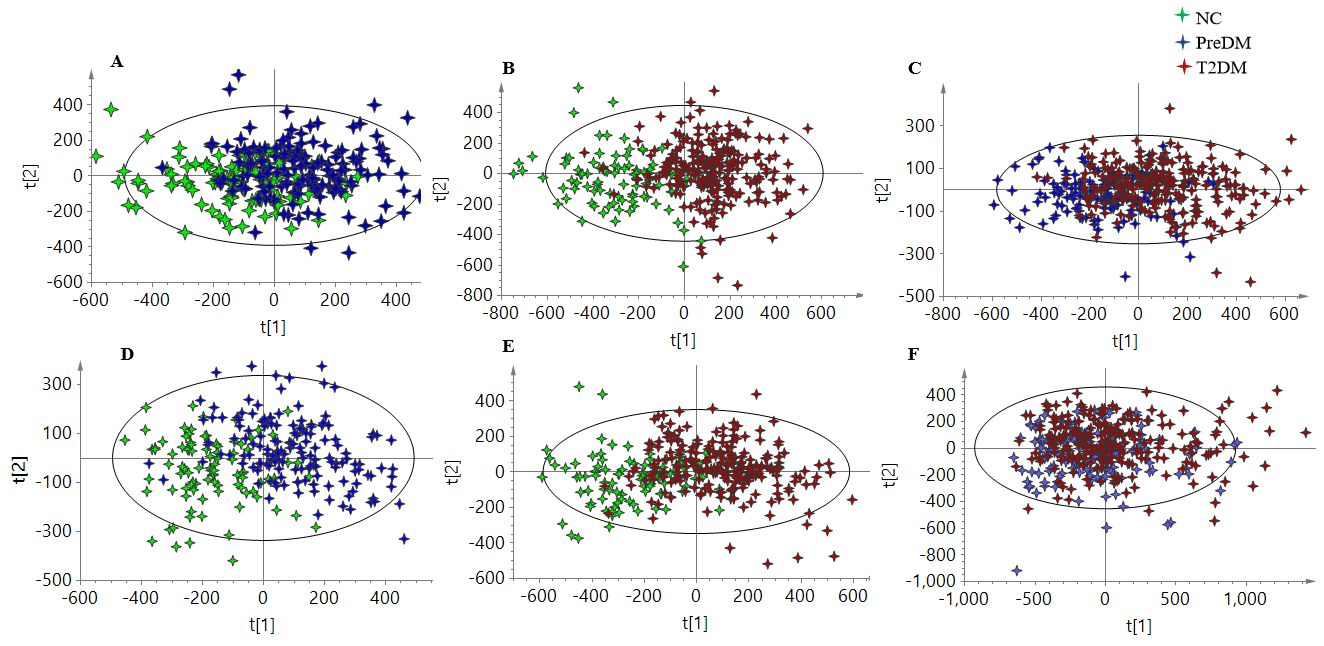


Figure S2. Score plots of orthogonal signal correction-partial least squares discriminant analysis (OSC-PLS-DA) for (A) PreDM vs. NC, (B) T2DM vs. NC, (C) T2DM vs. PreDM in the discovery set, and (D) PreDM vs. NC, (E) T2DM vs. NC, (F) T2DM vs. PreDM in the validation set, respectively.


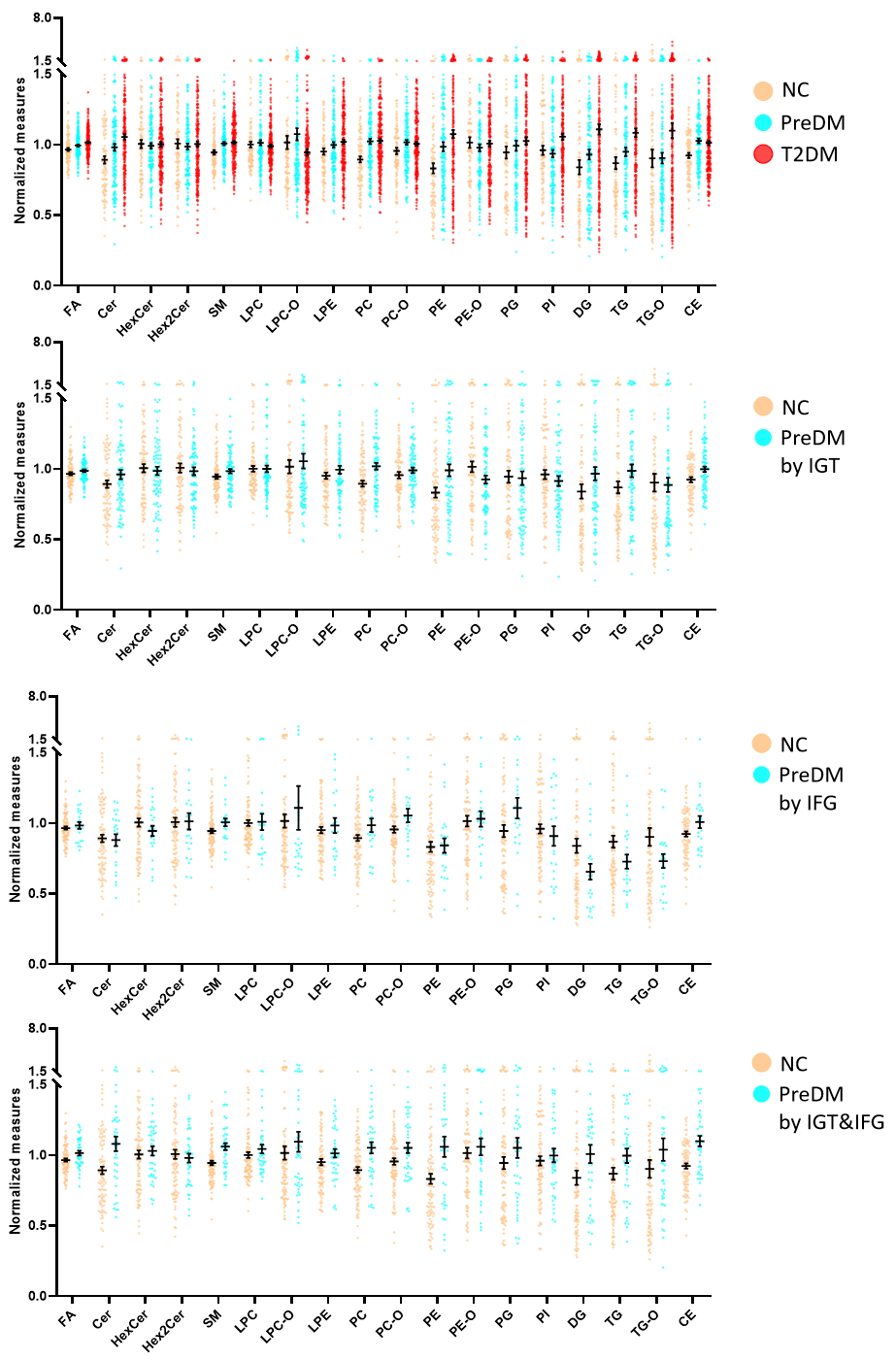


Figure S3. Mean normalised lipid (sub)species levels in T2DM, PreDM and NC by (a) any of the three criteria for PreDM, (b) IGT, (c) IFG and (d) IGT&IFG in the validation set. Data are expressed as means ± SEM.

Table S1. Retention time and ion pair information of lipid internal standards

| **Lipid IS** | **Precursor ions** | **Product ions** | **Q1** | **Q3** | **t*_R_*/ min** |
| --- | --- | --- | --- | --- | --- |
| FA 16:0-d3 | [M-H]^⁻^ | [M-H]^⁻^ | 258.3 | 258.3 | 1.41 |
| LPC 19:0 | [M+H]^⁺^ | [Pcho+H]^⁺^ | 538.4 | 184.1 | 1.45 |
| FA 18:0-d3 | [M-H]^⁻^ | [M-H]^⁻^ | 286.3 | 286.3 | 1.58 |
| SM (d18:1/12:0) | [M+H]^⁺^ | [Pcho+H]^⁺^ | 647.5 | 184.1 | 2.18 |
| PE (15:0/15:0) | [M+H]^⁺^ | [M+H-141]^⁺^ | 664.5 | 523.5 | 2.92 |
| PS (17:0/17:0) | [M-H]^⁻^ | [M-H-87]^⁻^ | 762.5 | 675.5 | 3.20 |
| DG (14:0/14:0) | [M+NH4]^⁺^ | [M+NH4-17-RCOOH]^⁺^ | 530.5 | 285.2 | 3.25 |
| Cer (d18:1/17:0) | [M+H]^⁺^ | [Sph backbone]^⁺^ | 552.5 | 264.3 | 3.72 |
| PE (17:0/17:0) | [M+H]^⁺^ | [M+H-141]^⁺^ | 720.6 | 579.5 | 3.91 |
| PC (19:0/19:0) | [M+H]^⁺^ | [Pcho+H]^⁺^ | 818.7 | 184.1 | 4.70 |
| TG (15:0/15:0/15:0) | [M+NH4]^⁺^ | [M+NH4-17-RCOOH]^⁺^ | 782.7 | 523.5 | 6.64 |
| TG 60:1-d5 | [M+NH4]^⁺^ | [M+NH4-17-RCOOH]^⁺^ | 995.9 | 666.9 | 8.35 |

Table S2. Key lipids: ORs of PreDM versus NC after adjusting for age, sex and BMI

| **Lipids** | **PreDM *vs.* NC** | | | | | |
| --- | --- | --- | --- | --- | --- | --- |
|  | **Discovery set** | | | **Validation set** | | |
|  | **OR(95%Cl)** | **Adjusted *p*** | **FC** | **OR(95%Cl)** | **Adjusted *p*** | **FC** |
| FA 20:2 | 1.518(1.115-2.066) | 0.008 | 1.14 | 1.582(1.134-2.206) | 0.007 | 1.12 |
| PC 35:2 | 1.399(1.015-1.928) | 0.040 | 1.14 | 1.401(0.997-1.967) | 0.052 | 1.12 |
| PC 32:0 | 1.374(1.006-1.877) | 0.046 | 1.11 | 1.898(1.344-2.681) | 0.000 | 1.11 |
| PC 35:3 | 1.372(0.988-1.904) | 0.059 | 1.17 | 1.654(1.170-2.339) | 0.004 | 1.20 |
| TG 56:8(22:6_X_X) | 1.343(0.951-1.897) | 0.094 | 1.33 | 1.367(0.996-1.876) | 0.053 | 1.30 |
| PC 40:6 | 1.329(0.971-1.819) | 0.076 | 1.12 | 1.998(1.366-2.923) | 0.000 | 1.17 |
| PC O-38:1 | 1.302(0.956-1.773) | 0.094 | 1.09 | 1.472(1.033-2.096) | 0.032 | 1.10 |
| PC 38:7 | 1.290(0.926-1.795) | 0.132 | 1.19 | 1.852(1.266-2.708) | 0.001 | 1.26 |
| PC 40:5 | 1.279(0.932-1.755) | 0.127 | 1.17 | 1.388(0.998-1.930) | 0.051 | 1.18 |
| PC 37:3 | 1.252(0.915-1.712) | 0.160 | 1.15 | 1.609(1.132-2.286) | 0.008 | 1.22 |
| FA 18:2 | 1.246(0.934-1.663) | 0.135 | 1.03 | 1.393(1.026-1.891) | 0.034 | 1.02 |
| PC 40:8_1 | 1.239(0.865-1.773) | 0.243 | 1.18 | 1.585(1.094-2.296) | 0.015 | 1.22 |
| SM 34:2 | 1.228(0.896-1.684) | 0.202 | 1.09 | 1.385(0.982-1.952) | 0.064 | 1.08 |
| PC 38:7 | 1.228(0.887-1.699) | 0.216 | 1.18 | 1.818(1.240-2.667) | 0.002 | 1.25 |
| PE 40:6 | 1.224(0.904-1.657) | 0.191 | 1.24 | 1.817(1.213-2.721) | 0.004 | 1.38 |
| PC 40:8_2 | 1.218(0.858-1.729) | 0.270 | 1.16 | 1.573(1.090-2.271) | 0.015 | 1.21 |
| PE 38:4 | 1.171(0.871-1.574) | 0.296 | 1.13 | 1.407(0.996-1.987) | 0.053 | 1.15 |
| PE 38:6 | 1.141(0.852-1.529) | 0.377 | 1.20 | 1.827(1.219-2.737) | 0.003 | 1.35 |
| TG 54:6(16:0_X_X) | 1.106(0.836-1.462) | 0.480 | 1.09 | 1.330(0.946-1.870) | 0.101 | 1.25 |
| PC 40:7 | 1.084(0.780-1.506) | 0.633 | 1.16 | 1.991(1.284-3.088) | 0.002 | 1.28 |
| TG 54:6(20:4_X_X) | 1.052(0.802-1.380) | 0.713 | 1.05 | 1.297(0.917-1.833) | 0.142 | 1.25 |
| TG 54:5(16:0_X_X) | 1.033(0.787-1.355) | 0.816 | 1.02 | 1.277(0.907-1.797) | 0.162 | 1.20 |

Table S3. Key lipids: ORs of T2DM versus NC after adjusting for age, sex and BMI

| **Lipids** | **T2DM *vs.* NC** | | | | | |
| --- | --- | --- | --- | --- | --- | --- |
|  | **Discovery set** | | | **Validation set** | | |
|  | **OR(95%Cl)** | **Adjusted *p*** | **FC** | **OR(95%Cl)** | **Adjusted *p*** | **FC** |
| SM 40:7 | 2.366(1.680-3.334) | 0.000 | 1.32 | 2.246(1.579-3.193) | 0.000 | 1.32 |
| FA 20:2 | 2.108(1.495-2.973) | 0.000 | 1.21 | 2.678(1.800-3.984) | 0.000 | 1.22 |
| SM 42:1 | 2.024(1.423-2.881) | 0.000 | 1.13 | 1.890(1.333-2.681) | 0.000 | 1.13 |
| PC 40:6 | 1.790(1.279-2.505) | 0.001 | 1.15 | 1.718(1.204-2.451) | 0.003 | 1.16 |
| SM 41:1 | 1.749(1.237-2.473) | 0.002 | 1.12 | 1.604(1.161-2.217) | 0.004 | 1.11 |
| SM 38:1 | 1.731(1.247-2.402) | 0.001 | 1.15 | 2.203(1.552-3.128) | 0.000 | 1.16 |
| TG 50:3(18:2_X_X) | 1.726(1.243-2.399) | 0.001 | 1.32 | 1.635(1.170-2.285) | 0.004 | 1.25 |
| PC 32:0 | 1.697(1.217-2.366) | 0.002 | 1.14 | 1.924(1.358-2.727) | 0.000 | 1.14 |
| TG 50:2(18:1_X_X) | 1.673(1.203-2.326) | 0.002 | 1.28 | 1.690(1.203-2.374) | 0.002 | 1.23 |
| SM 36:1 | 1.634(1.188-2.246) | 0.003 | 1.12 | 1.735(1.260-2.390) | 0.001 | 1.10 |
| TG 48:1(18:1_X_X) | 1.634(1.164-2.295) | 0.005 | 1.46 | 1.575(1.103-2.248) | 0.012 | 1.41 |
| SM 32:1 | 1.626(1.191-2.220) | 0.002 | 1.18 | 1.644(1.190-2.272) | 0.003 | 1.16 |
| TG 48:1(16:0_X_X) | 1.591(1.137-2.228) | 0.007 | 1.43 | 1.563(1.090-2.243) | 0.015 | 1.39 |
| TG 54:4(16:0_X_X) | 1.586(1.157-2.175) | 0.004 | 1.37 | 1.715(1.203-2.445) | 0.003 | 1.35 |
| TG 52:5(16:0_X_X) | 1.578(1.164-2.140) | 0.003 | 1.42 | 1.438(1.041-1.987) | 0.028 | 1.32 |
| Cer(d18:1/24:0) | 1.576(1.154-2.153) | 0.004 | 1.19 | 1.766(1.270-2.456) | 0.001 | 1.20 |
| TG 52:1(18:0_X_X) | 1.567(1.127-2.178) | 0.007 | 1.42 | 1.831(1.235-2.715) | 0.003 | 1.44 |
| TG 54:6(18:1_X_X) | 1.563(1.119-2.183) | 0.009 | 1.39 | 1.912(1.260-2.903) | 0.002 | 1.50 |
| TG 50:4(18:2_X_X) | 1.560(1.142-2.132) | 0.005 | 1.41 | 1.412(1.027-1.942) | 0.034 | 1.33 |
| SM 34:2 | 1.558(1.118-2.172) | 0.009 | 1.15 | 1.606(1.149-2.244) | 0.006 | 1.11 |
| TG 52:1(16:0_X_X) | 1.550(1.117-2.150) | 0.009 | 1.41 | 1.940(1.278-2.943) | 0.002 | 1.48 |
| TG 48:2(18:2_X_X) | 1.545(1.125-2.123) | 0.007 | 1.43 | 1.561(1.090-2.236) | 0.015 | 1.44 |
| FA 18:2 | 1.505(1.131-2.004) | 0.005 | 1.03 | 1.658(1.235-2.225) | 0.001 | 1.02 |
| TG 54:6(18:1_X_X) | 1.479(1.083-2.021) | 0.014 | 1.42 | 1.267(0.951-1.688) | 0.105 | 1.23 |
| TG 54:5(16:0_X_X) | 1.467(1.080-1.991) | 0.014 | 1.34 | 1.815(1.243-2.650) | 0.002 | 1.42 |
| TG 50:3(16:1_X_X) | 1.446(1.056-1.979) | 0.021 | 1.26 | 1.647(1.154-2.351) | 0.006 | 1.26 |
| TG 51:2(16:0_X_X) | 1.442(1.056-1.970) | 0.021 | 1.33 | 1.997(1.310-3.044) | 0.001 | 1.43 |
| SM 40:2 | 1.440(1.058-1.959) | 0.020 | 1.08 | 1.643(1.184-2.278) | 0.003 | 1.08 |
| PC 38:7_1 | 1.438(1.019-2.029) | 0.039 | 1.18 | 1.535(1.084-2.172) | 0.016 | 1.20 |
| TG 54:7(18:3_X_X) | 1.398(1.027-1.904) | 0.033 | 1.44 | 1.146(0.886-1.484) | 0.299 | 1.17 |
| TG 54:2(16:0_X_X) | 1.386(1.028-1.870) | 0.032 | 1.31 | 1.971(1.316-2.953) | 0.001 | 1.38 |
| PC 38:7_2 | 1.371(0.977-1.925) | 0.068 | 1.17 | 1.513(1.073-2.133) | 0.018 | 1.19 |
| TG 51:2(18:1_X_X) | 1.369(1.006-1.862) | 0.046 | 1.28 | 1.872(1.256-2.792) | 0.002 | 1.35 |
| TG 52:5(18:1_X_X) | 1.357(1.015-1.814) | 0.039 | 1.35 | 1.440(1.037-1.998) | 0.029 | 1.32 |
| TG 49:1(16:0_X_X) | 1.352(0.991-1.843) | 0.057 | 1.28 | 1.711(1.150-2.545) | 0.008 | 1.41 |
| TG 50:0(16:0_X_X) | 1.331(0.982-1.803) | 0.065 | 1.34 | 1.629(1.066-2.488) | 0.024 | 1.56 |
| TG 50:4(16:0_X_X) | 1.309(0.984-1.743) | 0.065 | 1.32 | 1.572(1.095-2.258) | 0.014 | 1.42 |
| TG 46:0(16:0_X_X) | 1.298(0.961-1.754) | 0.089 | 1.37 | 1.323(0.916-1.911) | 0.135 | 1.52 |
| TG 52:6(18:3_X_X) | 1.280(0.976-1.679) | 0.074 | 1.30 | 1.282(0.944-1.743) | 0.112 | 1.25 |
| TG 48:2(16:1_X_X) | 1.226(0.916-1.641) | 0.170 | 1.20 | 1.400(0.994-1.973) | 0.054 | 1.27 |
| TG 46:1(16:0_X_X) | 1.224(0.920-1.629) | 0.165 | 1.24 | 1.028(0.785-1.346) | 0.841 | 1.06 |
| TG 48:3(18:2_X_X) | 1.215(0.926-1.595) | 0.160 | 1.19 | 1.149(0.851-1.551) | 0.364 | 1.15 |
| TG 48:3(16:1_X_X) | 1.215(0.925-1.595) | 0.162 | 1.22 | 1.380(0.983-1.937) | 0.062 | 1.32 |
| TG 48:3(16:0_X_X) | 1.184(0.915-1.530) | 0.199 | 1.20 | 1.395(0.983-1.980) | 0.062 | 1.41 |
| TG 44:0(16:0_X_X) | 1.184(0.892-1.572) | 0.242 | 1.21 | 0.935(0.729-1.200) | 0.6 | 0.85 |
| TG 52:5(20:4_X_X) | 1.169(0.907-1.506) | 0.229 | 1.21 | 1.505(1.039-2.181) | 0.031 | 1.43 |
| TG 50:4(16:1_X_X) | 1.139(0.868-1.496) | 0.349 | 1.16 | 1.415(1.000-2.003) | 0.05 | 1.25 |
| PC 40:7 | 1.136(0.816-1.582) | 0.449 | 1.14 | 1.373(0.973-1.938) | 0.071 | 1.18 |
